# Orchiectomy as a result of ischemic orchitis after laparoscopic inguinal hernia repair: case report of a rare complication

**DOI:** 10.1186/1754-9493-1-3

**Published:** 2007-11-07

**Authors:** John B Moore, Erik A Hasenboehler

**Affiliations:** 1 Department of Surgery, Denver Health Medical Center, University of Colorado School of Medicine, 777 Bannock Street, Denver, CO 80204, USA; 2 Department of Orthopaedic Surgery, Denver Health Medical Center, University of Colorado School of Medicine, 777 Bannock Street, Denver, CO 80204, USA

## Abstract

**Background:**

Ischemic orchitis is an established complication after open inguinal hernia repair, but ischemic orchitis resulting in orchiectomy after the laparoscopic approach has not been reported.

**Case presentation:**

The patient was a thirty-three year-old man who presented with bilateral direct inguinal hernias, right larger than left. He was a thin, muscular male with a narrow pelvis who underwent bilateral extraperitoneal mesh laparoscopic inguinal hernia repair. The case was complicated by pneumoperitoneum which limited the visibility of the pelvic anatomy; however, the mesh was successfully deployed bilaterally. Cautery was used to resect the direct sac on the right. The patient was discharged the same day and doing well with minimal pain and swelling until the fourth day after surgery. That night he presented with sudden-onset pain and swelling of his right testicle and denied both trauma to the area and any sexual activity. Ultrasound of the testicle revealed no blood flow to the testicle which required exploration and subsequent orchiectomy.

**Conclusion:**

Ischemic orchitis typically presents 2–3 days after inguinal hernia surgery and can progress to infarction. This ischemic injury is likely due to thrombosis of the venous plexus, rather than iatrogenic arterial injury or inappropriate closure of the inguinal canal. Ultrasound/duplex scanning of the postoperative acute scrotum can help differentiate ischemic orchitis from infarction. Unfortunately, testicular torsion cannot be ruled out and scrotal exploration may be necessary. Although ischemic orchitis, atrophy, and orhiectomy are uncommon complications, all patients should be warned of these potential complications and operative consent should include these risks irrespective of the type of hernia or the surgical approach.

## Background

Testicular atrophy and necrosis are established and dreaded complications of inguinal hernia repair that surgeons may encounter regardless of their experience in the procedure[1]. Testicular atrophy is an uncommon complication of primary hernia repair. The current incidence is difficult to calculate as most literature that contains this data point predates laparoscopic repairs. For primary open inguinal hernioplasty testicular atrophy occurs in 0.5%, while for open recurrent hernioplasty the incidence can approach 5% [2-4]. Iles[5] reports a 1% incidence of testicular atrophy in 28,760 open inguinal hernia repairs, while Phillips[6] notes testicular problems including pain, swelling, and orchitis in 0.9% to 1.5% of laparoscopic inguinal hernia repairs. Ischemic orchitis was noted in a small number of these patients, but none resulted in testicular atrophy. Orchitis is thought to be more common after open procedures particularly associated with large indirect and recurrent inguinal hernias due to greater manipulation of the spermatic cord beyond the pubic tubercle and during dissection of the distal hernia sac[7].

Wantz[8] reported eleven (0.49%) incidences of ischemic orchitis with 2240 primary Shouldice hernioplasties of which only two (0.09%) resulted in testicular atrophy. All these cases were associated with scrotal indirect inguinal hernias with extensive cord dissection to remove the hernia sacs. Subsequently, he stopped excising the distal hernia sacs in 1409 patients with no occurrence of ischemic orchitis.

## Case presentation

**Figure 1:**
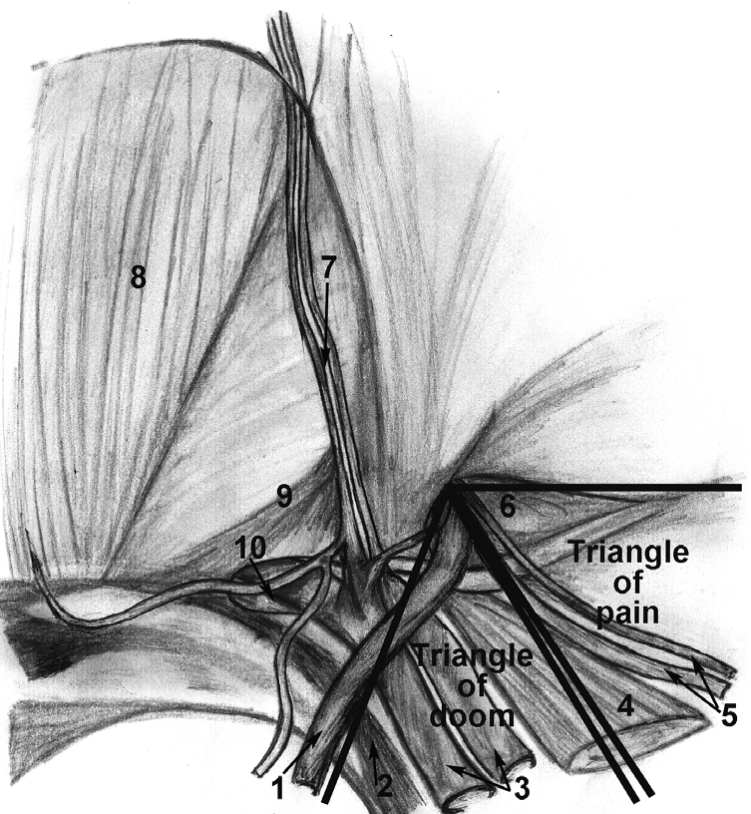
Anatomical representation of the internal inguinal hernia wall (1.Vas Deference, 2.Ligament of Cooper, 3.External Iliac Vessels, 4.Psoas Muscle, 5.Testicular Vessels, 6.Indirect Inguinal Hernia, 7.Inferior Epigastric Vessels, 8.Rectus Abdominis Muscle, 9.Inguinal Falx, 10.Direct Inguinal Hernia).

**Figure 2:**
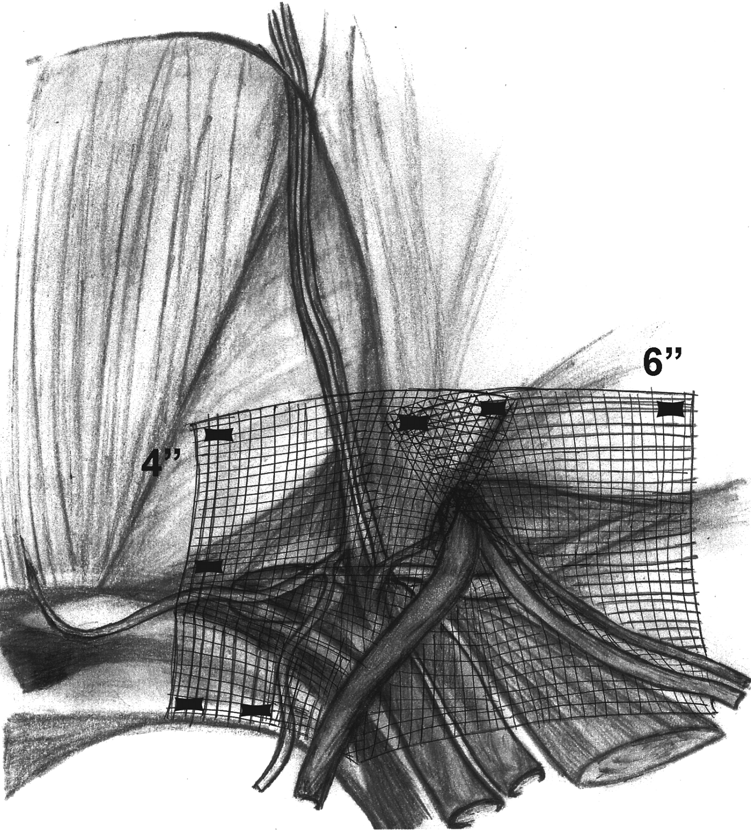
The first mesh (4×6 inches) with vertical-slit is placed beneath the cord structures and attached with sutures avoiding the "triangle of doom" and "triangle of pain".

**Figure 3:**
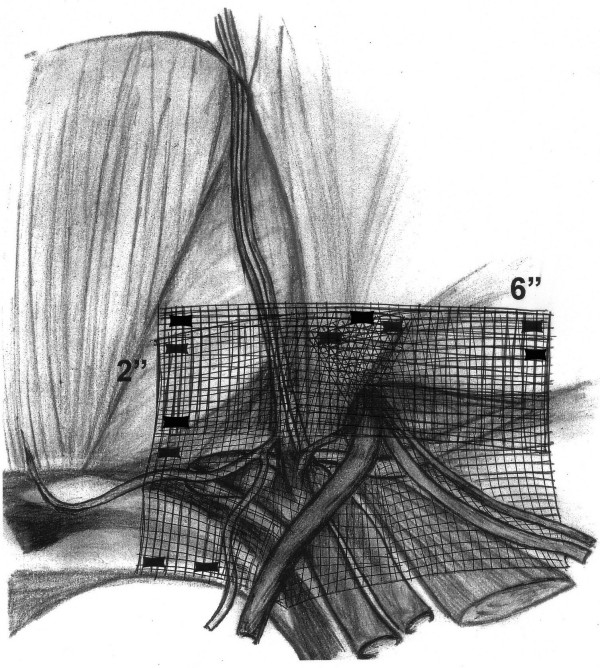
A second overlay mesh (2×6 inches) is placed over the new internal ring created by the slit and tacked in a similar manner to the first mesh.

We evaluated a thin, muscular thirty-three year-old man with symptomatic bilateral direct inguinal hernias. We noted that he had a narrow pelvis and that the right hernia was greater than the left. Both were reducible and occasionally painful while straining, but no extension into the scrotum was noted. He had no previous abdominal surgery or pelvic trauma. He underwent extraperitoneal laparoscopic bilateral mesh hernioplasty. During the placement of the dissection balloon, the peritoneum was violated, causing a pneumoperitoneum. The peritoneal tear was repaired using endo-loops. Despite trendelenburg positioning, the pneumoperitoneum limited the visibility in the patient's narrow pelvis. The right hernia defect was a moderate-sized direct. After identifying the cord structures and epigastric vessels (Fig. 1), a vertical-slit flexible mesh was placed beneath the cord structures and tacked into place (Fig. 2) avoiding the "triangle of doom" (iliac vessels) and the "triangle of pain" (genitofemoral nerve/lateral cutaneous femoral nerve) (Fig. 1). A second overlay piece of mesh was placed over the new internal ring created by the slit and tacked in a similar manner (Fig. 3). The right direct hernia sac was removed with cautery excision to minimize post-op seroma. The left side was repaired in a similar manner. The CO_2 _was evacuated as the peritoneum was observed to lay over the meshes completely.

The patient went home and was recovering well, with minimal edema and lessening pain until the fourth post-operative day. He was awakened by progressive pain and swelling of his right testicle. He was evaluated with doppler ultrasound and found to have a heterogeneically hypoechoic right testicle with no identifiable arterial flow consistent with infarction. Immediate urological consultation considered ischemic orchitis secondary to the inguinal hernia repair or less likely testicular torsion. Exploration revealed a necrotic appearing right testicle without signs of torsion. The tunica vaginalis was opened followed by a deep testicular incision that revealed no bleeding. The right testicle was considered nonviable; therefore, right orchiectomy was performed. Thrombus was noted grossly within the spermatic venous complex. Pathology revealed infarcted testicular parenchyma.

## Conclusion

Testicular ischemia and necrosis after laparoscopic surgery is an uncommon complication that is reported rarely in the literature. It is thought to be due to acute thrombosis of the pampiniform venous plexus rather than arterial injury, as there is collateral arterial flow to the testis from the inferior epigastric, vesical, prostatic and scrotal arteries[7]. Even in cases where the spermatic cord is purposely ligated, one-third of the testes will not become ischemic[9].

The patient was a good candidate for laparoscopic preperitoneal hernioplasty with bilateral primary direct inguinal hernias and no previous abdominal surgery or pelvic trauma. The most likely cause of the right testicular infarction in our case was the cautery excision of the right direct hernia sac that led to venous injury. This maneuver was compromised by less than optimal visualization secondary to the narrow male pelvis aggravated by the pneumoperitoneum. The use of the vertical slit mesh technique has been reported to increase the risk of recurrent hernias, especially in indirect types[10]. As previously described, we use the vertical slit mesh (4×6") with a second overlay (2×6") to reinforce the newly created internal ring (Fig. 2, 3)[11].

The need for right orchiectomy in this 33 year old man presenting with an acute scrotum 4 days after laparoscopic inguinal hernioplasty should be addressed. The testicular ultrasound/duplex scan indicated a diagnosis of infarcting right testicle. Despite the more likely diagnosis of right testicular infarction secondary to the recent laparoscopic inguinal hernioplasty, torsion of the testicle had to be considered. Torsion of the testicle has been reported in 26 – 39% of cases in men over age 21; while up to 10% occur in men over age 30[12,13]. The finding of a necrotic testicle without torsion was confirmed by lack of any bleeding in a deep incision of the testicular tissue within 10 minutes and led to orchiectomy. Leaving a necrotic right testicle in situ could have impacted subsequent fertility by inducing autoimmunization against spermatozoa[14].

This patient was not informed of the rare possibility of orchiectomy as a complication of laparoscopic preperitoneal hernia repair. Considering the magnitude of this complication, albeit not previously reported, we recommend a frank discussion of possible orchitis, atrophy, or rarely orchiectomy. Despite the rarity of this complication or possibility of frightening the patient unnecessarily, the medical legal climate mandates this counseling.

We would add that in preperitoneal laparoscopic repairs, if the preperitoneal space cannot be properly visualized, one should consider conversion to an intraperitoneal laparoscopic or open approach. We also caution against the use of cautery in proximity to tissues that could lead to venous thrombosis of the cord structures. Finally, the diagnosis of evolving testicular ischemia should be rapidly sought via doppler ultrasound or with nuclear imaging so as to limit injury to the testicle[15,16].

## Competing interests

The author(s) declare that they have no competing interests.

## Authors' contributions

**JM: **Wrote and edited the manuscript.

**EH: **Edited the final version of the manuscript and provided the graphic art work for figures 1, 2 and 3.

All authors read and approved the final manuscript.

## References

[B1] Koontz AR (1965). Atrophy of the Testicle as a Surgical Risk. Surgery, gynecology & obstetrics.

[B2] Wantz GE (1984). Complications of inguinal hernial repair. The Surgical clinics of North America.

[B3] Reid I, Devlin HB (1994). Testicular atrophy as a consequence of inguinal hernia repair. The British journal of surgery.

[B4] Skandalakis JE, Skandalakis LJ, Colborn GL (1996). Testicular atrophy and neuropathy in herniorrhaphy. The American surgeon.

[B5] Iles JD (1965). Specialisation in Elective Herniorrhaphy. Lancet.

[B6] Phillips EH, Arregui M, Carroll BJ, Corbitt J, Crafton WB, Fallas MJ, Filipi C, Fitzgibbons RJ, Franklin MJ, McKernan B (1995). Incidence of complications following laparoscopic hernioplasty. Surgical endoscopy.

[B7] Fong Y, Wantz GE (1992). Prevention of ischemic orchitis during inguinal hernioplasty. Surgery, gynecology & obstetrics.

[B8] Wantz GE (1986). Testicular atrophy as a sequela of inguinal hernioplasty. International surgery.

[B9] Heifetz CJ (1971). Resection of the spermatic cord in selected inguinal hernias. Twenty years of experience. Arch Surg.

[B10] Korman JE, Hiatt JR, Feldmar D, Phillips EH (1997). Mesh configurations in laparoscopic extraperitoneal herniorrhaphy. A comparison of techniques. Surgical endoscopy.

[B11] Felix EL, Michas C (1993). Double-buttress laparoscopic herniorrhaphy. Journal of laparoendoscopic surgery.

[B12] Lee LM, Wright JE, McLoughlin MG (1983). Testicular torsion in the adult. The Journal of urology.

[B13] Witherington R, Jarrell TS (1990). Torsion of the spermatic cord in adults. The Journal of urology.

[B14] Arda IS, Ozyaylali I (2001). Testicular tissue bleeding as an indicator of gonadal salvageability in testicular torsion surgery. BJU international.

[B15] Holloway BJ, Belcher HE, Letourneau JG, Kunberger LE (1998). Scrotal sonography: a valuable tool in the evaluation of complications following inguinal hernia repair. J Clin Ultrasound.

[B16] Akin EA, Khati NJ, Hill MC (2004). Ultrasound of the scrotum. Ultrasound quarterly.

